# Bicyclol attenuates high fat diet-induced non-alcoholic fatty liver disease/non-alcoholic steatohepatitis through modulating multiple pathways in mice 

**DOI:** 10.3389/fphar.2023.1157200

**Published:** 2023-03-17

**Authors:** Jingyi Wu, Shu Jia, Benhong Xu, Xiaokun Yao, Jingping Shao, Jianzuo Yao, Danwei Cen, Xiaomin Yao

**Affiliations:** ^1^ Faculty of Pharmacy, Zhejiang Pharmaceutical University, Ningbo, China; ^2^ Shenzhen Key Laboratory of Modern Toxicology, Shenzhen Medical Key Discipline of Health Toxicology, Shenzhen Center for Disease Control and Prevention, Shengzhen, Guangdong, China; ^3^ Department of Hepatobiliary and Pancreatic Surgery, Li Huili Hospital Affiliated to Ningbo University, Ningbo, China

**Keywords:** bicyclol, NAFLD, NASH, proteomics, multiple pathways

## Abstract

**Introduction:** The pathological progression of non-alcoholic fatty liver disease (NAFLD) is driven by multiple factors, and non-alcoholic steatohepatitis (NASH) represents its progressive form. In our previous studies, we found that bicyclol had beneficial effects on NAFLD/ NASH. Here we aim to investigate the underlying molecular mechanisms of the bicyclol effect on NAFLD/NASH induced by high-fat diet (HFD) feeding.

**Methods:** A mice model of NAFLD/NASH induced by HFD-feeding for 8 weeks was used. As a pretreatment, bicyclol (200 mg/kg) was given to mice by oral gavage twice daily. Hematoxylin and eosin (H&E) stains were processed to evaluate hepatic steatosis, and hepatic fibrous hyperplasia was assessed by Masson staining. Biochemistry analyses were used to measure serum aminotransferase, serum lipids, and lipids in liver tissues. Proteomics and bioinformatics analyses were performed to identify the signaling pathways and target proteins. Data are available *via* Proteome X change with identifier PXD040233. The real-time RT-PCR and Western blot analyses were performed to verify the proteomics data.

**Results:** Bicyclol had a markedly protective effect against NAFLD/NASH by suppressing the increase of serum aminotransferase, hepatic lipid accumulation and alleviating histopathological changes in liver tissues. Proteomics analyses showed that bicyclol remarkably restored major pathways related to immunological responses and metabolic processes altered by HFD feeding. Consistent with our previous results, bicyclol significantly inhibited inflammation and oxidative stress pathway related indexes (SAA1, GSTM1 and GSTA1). Furthermore, the beneficial effects of bicyclol were closely associated with the signaling pathways of bile acid metabolism (NPC1, SLCOLA4 and UGT1A1), cytochrome P450-mediated metabolism (CYP2C54, CYP3A11 and CYP3A25), biological processes such as metal ion metabolism (Ceruloplasmin and Metallothionein-1), angiogenesis (ALDH1A1) and immunological responses (IFI204 and IFIT3).

**Discussion:** These findings suggested that bicyclol is a potential preventive agent for NAFLD/NASH by targeting multiple mechanisms in future clinical investigations.

## 1 Introduction

Non-alcoholic fatty liver disease (NAFLD) is the most common metabolic syndrome worldwide (Meroni et al., 2020; Fu et al., 2022). The progression of NAFLD from the pure lipid accumulation to non-alcoholic steatohepatitis (NASH) further culminates into fibrosis, cirrhosis, and hepatocellular cancer (Gehrke and Schattenberg, 2020; Wei et al., 2021). Although some studies have identified the drivers and pathogenesis of NAFLD, approved treatment and specific drug are still not available and need to be urgently developed.

It is generally believed that NAFLD is not a single-factor disorder, and the progression of NAFLD/NASH is driven by multiple targets, such as imbalance of inflammation, oxidative stress, immunological responses, bile acid metabolism, angiogenesis (Lei et al., 2021; Tilg et al., 2021; Xue et al., 2021). The deactivation of signaling molecules (TGF-β1 and MKK3/MAPK) related to inflammation and oxidative stress could alleviate NASH progression (Ahmed et al., 2022; Ding et al., 2022). Single-cell RNA sequencing revealed the activation of liver Kupffer cells beyond their typical inflammatory effect in the pathogenesis of NAFLD/NASH (Barreby et al., 2022). The dysfunction of bile acid metabolism was closely linked to NAFLD. Further, the activator of bile acid receptors, such as S1PR2, provided a novel NAFLD therapy (Xue et al., 2021). Research evidences explained the role of angiogenesis triggered by inflammation in NAFLD pathogenesis and highlighted potential of targeting VEGF to treat NAFLD (Lei et al., 2021). Ideally, a therapeutic agent should have the capacity to modulate multiple pathways participating in the NAFLD/NASH progression.

Bicyclol is a versatile biphenyl compound widely applied to treat chronic hepatitis in China. A systematic meta-analysis presented the evidence of bicyclol treatment for improving liver function and serum lipid biomarkers in patients with NAFLD, predicting that bicyclol might be an alternative agent for NAFLD (Li et al., 2020). The quality of evidence suggested that bicyclol administration significantly attenuated high-fat diet (HFD)-induced activation of inflammatory-mediated signaling pathway (MAPKs/NF-κB) and gluconeogenesis/insulin signaling pathway (Akt, PGC-1α and PEPCK) (Li H et al., 2021; Zhao W et al., 2021). Pharmacological studies displayed that bicyclol had obvious protective effects against hepatocyte injury caused by tetracycline, alcoholic and non-alcoholic fatty liver associated with cell apoptosis, endoplasmic reticulum (ER), and oxidative stress (Yao et al., 2016; Jia et al., 2022). Not only that, bicyclol intervention attenuated hepatocyte injury by moderating bile acid-mediated pathways (α-MCA/β-MCA) and regulating autophagy-related pathways (HMGB1/p62/Nrf2) (Zhao J et al., 2021; Zhao et al., 2022). Extensive pharmacological properties highlight the potential of bicyclol as a therapeutic agent of NAFLD.

Although several studies identified the molecular mechanisms underlying the protective effect of bicyclol on NAFLD/NASH, most of the studies focused on only one mechanism and were unable to systematically elucidate multiple mechanisms in NAFLD/NASH pathological process and the beneficial effect of bicyclol. Thus, further studies are needed to comprehensively delineate its molecular mechanisms and targets of bicyclol to explore bicyclol as a significantly potent drug for treating NAFLD/NASH.

In this research, we investigated the molecular mechanisms and signaling pathways of bicyclol attenuating NAFLD/NASH using a Tandem Mass Tag (TMT) proteomics approach, bioinformatics analyses and molecular biological technology. This study aimed to comprehensively characterize the multiple molecular mechanisms of NAFLD/NASH progress improved by bicyclol and to explore the potential target and pathway covering multiple pathogeneses of NAFLD/NASH.

## 2 Materials and methods

### 2.1 Animal population and study design

All the male ICR mice (8–9 weeks, weighing 21–26 g) were obtained from the Zhejiang Academy of Medical Sciences (Hangzhou, China). The animal experiments followed the experimental guidelines and internationally recognized principles in the animal care and were authorized by the Laboratory Animal Ethics Committee of Zhejiang Pharmaceutical University (Ningbo, China).

The 30 ICR mice were randomly categorized into normal group, model group and bicyclol group (10 mice in each group). Mice in both the model and bicyclol groups were fed with the HFD (including 40% fat and 1.25% cholesterol; Catalog No. D12108C), and the normal group mice were fed with a standard diet. Besides, the mice in the bicyclol group were given bicyclol (200 mg/kg) diluted in 0.5% carboxymethyl cellulose *via* oral gavage twice daily. The body weights of mice were monitored every other day. After feeding for 8 weeks, the liver tissues and blood samples of all the ICR mice were amassed for the follow-up experiments after 12 h diet deprivation.

### 2.2 Biochemistry analyses

The liver tissues and blood samples for biochemical analyses were obtained after 8 weeks of treatment. Serum alanine transaminase (ALT) and aspartate aminotransferase (AST) levels and liver tissue triglyceride (TG) and total cholesterol (CHO) contents were measured using assay kits (Nanjing Jiancheng, Catalog No. C009–3 and C010-3; Catalog No. A110–2 and A111-2) according to the manufacturer’ procedures.

### 2.3 Histopathology

The liver tissues were fixed in 4% formaldehyde solution and then embedded in paraffin. The 5-μm-thick slides were cut to perform hematoxylinandeosin (H&E) staining and Masson’s staining. The collagen volume fractions (CVFs) were analyzed by ImageJ software.

### 2.4 Proteomic analyses using TMT

All the tissues samples from the liver of mice were prepared based on the procedure described in the previous method (Guo et al., 2018). Ultrasonic homogenizer was used to lyse liver tissues (4 mice in each group) in ice-cold phosphate-buffered saline (PBS, pH8.0) including 8 M urea and 1× cocktail (Roche, IN, United States). After centrifuging, the concentrations were quantified using a NanoDrop spectrophotometer (Thermo Fisher Scientific, NJ, United States). Then, 100 μg of pooled proteins was treated with 10 mM dithiothreitol and incubated with 25 mM iodoacetamide. Subsequently, the protein samples digested with 1 g/L trypsin at 37 °C for 24 h, and further suspended in 1% 50 μL of formic acid (FA). The treated samples were centrifuged, and then the reversed phase column (Waters, OasisHLB; MA, United States) was used to further desalt the supernatants. After drying, the peptides were diluted with triethylammonium bicarbonate and then labelled using TMT reagents (0.8 mg TMT dissolved in 99.9% 40 μl acetonitrile) at room temperature for 1 h s.The labelled peptides were fragmented based on the method described in the previous method (Gokce et al., 2011). TMT-labeled peptides were loaded onto the Xbridge peptide BEH300 C18 column for HPLC (Ultimate 3000 UHPLC; Thermo Fisher Scientific, MA, United States) separation. The fragments were collected, dried, and suspended in 20 μL of 0.1% FA for liquid chromatography (LC)/mass spectrometry (MS) analyses.

An analytical capillary column (Upchurch, Oak Harbor, WA, United States) packed with C18 silica resin (Varian, Lexington, MA, United States) was used to separate peptides by gradient elution. Xcalibur 2.1.2 software was used to manipulate a Thermo Q Exactive mass spectrometer (Thermo Scientific, NJ, United States) in a data-dependent acquisition mode. After a single full-scan mass spectrum in Orbitrap, 10 data-dependent MS/MS scans were perfomed at 27% normalized collision energy (higher-energy C-trap dissociation, HCD).

Proteome Discoverer 2.1 software was used to compare the MS/MS spectra from each LC-MS/MS run with the UniProt mouse FASTA database (released on 19 June 2016). The recommended search criteria had be altered as follows: two missed cleavages allowed; full tryptic specificity required; oxidation (methionine, M); static modifications, carbamidomethylation and TMT plex (lysine [K] and any N-terminal); fragment ion mass tolerance, 20 mmu for all MS2 spectra acquired; precursor ion mass tolerances, 20 ppm for all MS acquired on an Orbitrap mass analyzer. The reporter ion intensities per peptide was used to quantify protein relatively based on manufacturer’s instructions. Protein ratio variability represented quantitative accuracy. The mass spectrometry proteomics data have been deposited to the ProteomeXchange Consortium *via* the PRIDE [1] partner repository with the dataset identifier PXD040233 and 10.6019/PXD040233.

### 2.5 Bioinformatics analyses

The proteins with a log_2_-transformed ratio of < −0.4 or >0.4 and *p* < 0.05 were determined as differentially expressed proteins between two comparative groups: model *versus* normal and bicyclol *versus* model. The functional classifications, single pathway enrichment, and gene ontology (GO) term analyses on the differentially expressed proteins were performed by bioinformatics method. GO enrichment analyses identified proteins involved in cell component, biological process, and molecular function using the Database for Annotation, Visualization, and Integrated Discovery (DAVID) bioinformatics resource. The Kyoto Encyclopedia of Genes and Genomes (KEGG) pathway was applied to analyze signaling pathways of the differentially expressed proteins. A corrected *p*-value < 0.05 indicated a statistically significant difference.

### 2.6 Real-time reverse transcription–quantitative polymerase chain reaction analyses

The total RNAs of liver tissues were easily extracted using an RNA isolation reagent (Nanjing Vazyme, Catalog No. R701) following the manufacturer’s protocol. The cDNA was reverse transcribed from the RNA using HiScript III All-in-one RT SuperMix (Nanjing Vazyme, Catalog No. R333). The real-time reverse transcription–quantitative polymerase chain reaction (RT-qPCR) was performed using ChamQ SYBR Color qPCR Master Mix (Nanjing Vazyme, Catalog No. Q421-02) with 40 cycles of 95 °C for 10 s and 60 °C for 30 s, followed by 95 °C for 15 s and 60 °C for 60 s using RT-qPCR System machine (LightCycler480; Roche, Switzerland). The relative expression levels of target genes were calculated by employing 2^−ΔΔCT^ method. The sequences of primers used for amplifying the target genes are listed in Table 1.

**TABLE 1:** Polymerase chain reaction primers used in the qPCR

Gene	Description	Primer sequences	GenBank^TM^accession no.
β-ACTIN	Beta-actin	Forward AGG​CAT​TGC​TGA​CAG​GAT​G	NM_133360
		Reverse TGC​TGA​TCC​ACA​TCT​GCT​GG	
GSTM1	Glutathione S-transferase Mu 1	Forward AATCTTCTCTTCTGTCTC	NM_000561.4
		Reverse AACGCCATCTTGTGCTAC	
GSTA1	Glutathione S-transferase A1	Forward AGGTTGCTGATTCTGGTT	NM_001319059.2
		Reverse TGG​TGG​AAC​TTC​TCT​ACT​AC	
RDH11	Retinol dehydrogenase 11	Forward AAGCCACCACATCCATAT	NM_001252650.2
		Reverse CAC​ATA​GGA​GTC​AAC​CAC​TT	
SAA1	Serum amyloid A-1 protein	Forward ATC​ACC​GAT​GCC​AGA​GAG​A	NM_000331.6
		Reverse GATCAGCCAGCGAGTCCT	
FTH1	Ferritin heavy chain	Forward TATTCCGCCAAGCCAGAT	NM_002032.3
		Reverse GCAGCCACATAACCAGAG	
CP	Ceruloplasmin	Forward CGG​CAA​TGT​AGT​AGT​GTC​TA	NM_000096.4
		Reverse CCAGGTCCAGGAGTGTAA	
SLCO1A4	Solute carrier organic anion transporter family member 1A4	Forward CCA​TTA​TCC​TGT​AAT​CCT​TCC	NM_001355577.2
		Reverse AGA​TGA​CCT​GAC​CAT​AAC​TC	
SLC22A7	Solute carrier family 22 member 7	Forward CAT​CCA​GCC​ACT​CTA​ACT​C	NM_144856.2
		Reverse GCCTCGGTCAACTACATC	
UGT1A1	UDP-glucuronosyltransferase 1–1	Forward CCA​CAA​TTC​CAT​GTT​CTC​C	NM_000463
		Reverse ATCAACTGCCTTCACCAA	
NPC1	Niemann-Pick C1 protein	Forward AAGCCAACACCACAATCC	NM_000271.5
		Reverse GCAGCCACATAACCAGAG	
ABCC3	ATP-binding cassette, sub-family C member 3	Forward GCTCTCGTCCACATACAC	NM_001363187.1
		Reverse CCA​TCT​CCA​CCT​TCA​TCT​G	
CYP2C54	Cytochrome P450 2C54	Forward AAC​ACG​AGG​CAC​TTC​TCA​CT	NM_206537
		Reverse GAA​CAC​GGT​CCT​CAA​TGC​TC	
CYP2C70	Cytochrome P450 2C70	Forward TGC​TGT​GCT​GCA​TGA​GAT​TC	NM_145499
		Reverse TGC​CCT​TGG​GAA​TGT​GGT​AT	
CYP51	Lanosterol 14-alpha demethylase	Forward ATG​GTA​CTT​CTG​GGC​TTG​CT	NM_020010
		Reverse AGG​TAG​ACG​AGG​CTG​AGA​G	
CYP3A25	Cytochrome P450 3A25	Forward TGGTGAAGGTTGGAGACA	NM_019792.2
		Reverse GGA​TGA​AGA​ATG​GAA​GAG​A	
CYP3A11	cytochrome P450 3A11	Forward CTC​AAG​TCT​ATT​AGC​AAT​GG	NM_007818.3
		Reverse GAT​GGA​ATA​CCT​GGA​TAT​GG	
CYP2A5	Cytochrome P450 2A5	Forward TGT​AGT​CAG​CAC​CAA​GTT​C	NM_007812.4
		Reverse CTC​CTT​CCT​CAT​CCG​AAT​G	
IFIT3	Interferon-induced protein with tetratricopeptide repeats 3	Forward ACT​CCA​TCG​TTA​ATC​GTC​TC	NM_010501.2
		Reverse ACA​GTG​AAC​AAC​AGT​CCT​C	
MT1	Metallothionein-1	Forward CTGGGCACATTTGGAGCA	NM_013602.3
		Reverse GCACCTCCTGCAAGAAGA	
IFI204	Interferon-activable protein 204	Forward CTG​AAT​CGT​GGT​GTA​TTG​C	NM_008329
		Reverse GCT​GAG​AGG​ACT​TGA​ATG​T	
HPGD	15-hydroxyprostaglandin dehydrogenase	Forward CATCACACTGGACGAACA	NM_008278.2
		Reverse AGC​ATT​GGT​GGA​TTG​GAA​T	
PCK1	phosphoenolpyruvate carboxykinase	Forward TCG​CAG​ATG​TGG​ATA​TAC​TC	NM_011044.3
		Reverse CAGGCAGTGAGGAAGTTC	
ALDH1A1	Aldehyde dehydrogenase family 1, subfamily A1	Forward TTA​CCA​CGC​CAT​AGC​AAT​T	NM_000689.5
		Reverse AGC​CAT​AAC​AAT​CTC​CTC​TG	

### 2.7 Western blot analyses

The RIPA lysis buffer (Beijing Bioshap, Catalog No. BL509A) was used to lyse liver samples. After centrifugation at 12,000g and 4 °C for 15 min, the supernatants were collected and determined using a BCA protein assay kit (Bioshap, Catalog No. BL521A). Then, 50 μg of each protein sample was dissolved in 10% SDS-PAGE solution and electrophoresed. All the protein samples were transferred to polyvinylidene fluoride membranes (Germany Merck, Catalog No. ISEQ00010). The target proteins in transfer membranes were probed using primary antibodies as follows: serum amyloid A-1 protein (SAA1) (1:800, Wuhan Proteintech, Catalog No. 25467-1-AP), uridine 5′-diphospho-glucuronosyltransferase 1–1 (UGT1A1) (1:1,200, Wuhan Proteintech, Catalog No. 23495-1-P), lanosterol 14-alpha demethylase (CYP51) (1:1,000, Wuhan Proteintech, Catalog No. 13431-1-AP), phosphoenolpyruvate carboxykinase (PCK1) (1:1,000, Shanghai Abcam, Catalog No. ab70358), and GAPDH (1:2000, Wuhan Proteintech, Catalog No. 60004-1-lg). After washing, the transfer membranes were further incubated with HRP-conjugated secondary antibodies and developed using ECL reagents. Finally, the membranes were exposed to a fluorescent gel imaging system (Amersham Imager 680; GE, Japan) for appropriate time durations to acquire the band images. The quantitative measures of the band intensities were analyzed using the IQTL 7.0 software.

### 2.8 Statistical analyses

The GraphPad 7.0 software was used to perform statistical analyses of the experimental data. All the values were expressed as the mean ± standard deviation. All the data were evaluated by the Tukey *t*-test and one-way analysis of variance using SPSS Statistics 22.0 software. A *p*-value <0.05 indicated a statistically significant difference.

## 3 Results

### 3.1 Effects of bicyclol on the histopathological and blood biochemistry in HFD-fed mice

**FIGURE 1:**
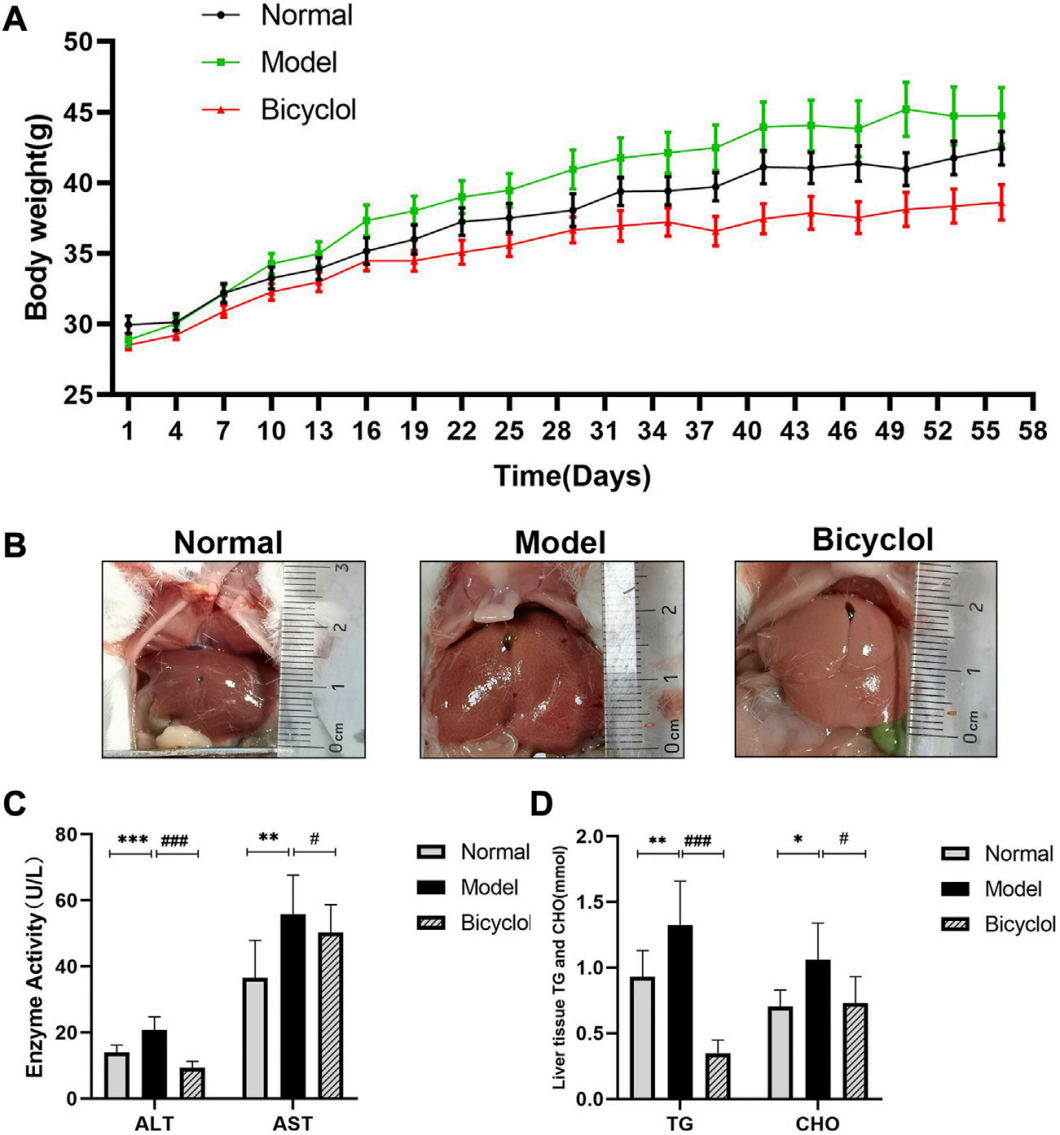
Effects of bicyclol on body weight and biochemical indexes of mice fed with HFD. **(A)** Changing of mice weight during feeding period of 8 weeks (*n* = 10). **(B)** Representative image of liver. **(C)** Analyses of liver transaminase (*n* = 9). **(D)** Analyses of TG and CHO in liver tissues (*n* = 9). *p < 0.05 vs Normal, **p < 0.01 vs Normal, ***p < 0.001 vs Normal, #*p* < 0.05 vs Model, ###*p* < 0.001 vs Model.

As shown in Figure 1A, HFD significantly increased body weight of mice after 8 weeks in the model group compared with the normal group. Continuous feeding with HFD promoted the weight gain of mice, which was dramatically reduced after bicyclol treatment. The feeding with HFD significantly increased liver volume with a more prominent fat accumulation in the model group compared with the normal group, which was alleviated by bicyclol treatment (Figure 1B). HFD feeding also significantly increased ALT and AST levels in serum, which were reduced after bicyclol treatment (Figure 1C). Furthermore, bicyclol treatment significantly attenuated total CHO and TG levels in the liver tissues, which were increased by HFD feeding (Figure 1D).

**FIGURE 2:**
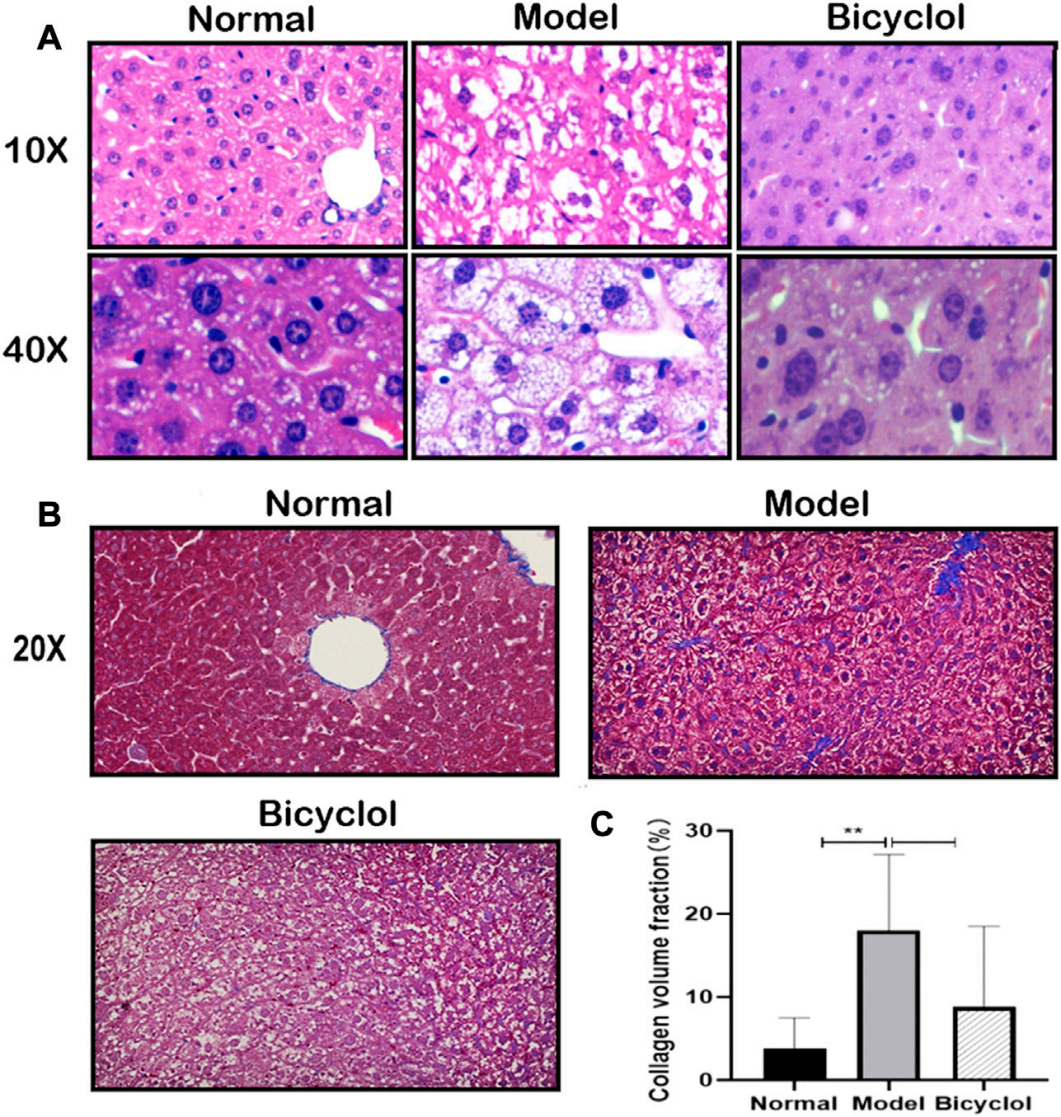
Effect of bicyclol on HFD feeding induced fatty and fibrotic liver. **(A)** Liver representative pathology images of H&E staining (scalebar, 100um for ×10magnification, 20p.m. for ×40magnification). **(B)** Liver representative images of Masson staining (scalebar, 40p.m. for ×20magnification). **(C)** CVF in the liver sections (n = 6).**p < 0.01 vs Normal.

The histological analysis of the liver tissues indicated that HFD feeding induced NAFLD/NASH after 8 weeks. As shown in Figure 2A, hepatocellular ballooning and steatosis were observed in the liver tissue of mice fed with HFD, using H&E staining, which were markedly alleviated by bicyclol treatment. Masson staining was observed with blue collagen fibers in the liver sections, which revealed that bicyclol significantly alleviated HFD-induced hepatic fibrous hyperplasia (Figure 2B). CVF in the liver sections of HFD mice was markedly higher in the model group than in the normal group, whereas bicyclol treatment reversed these increased CVFs (Figure 2C).

### 3.2 Bioinformatics analyses for the changed proteins

**FIGURE 3:**
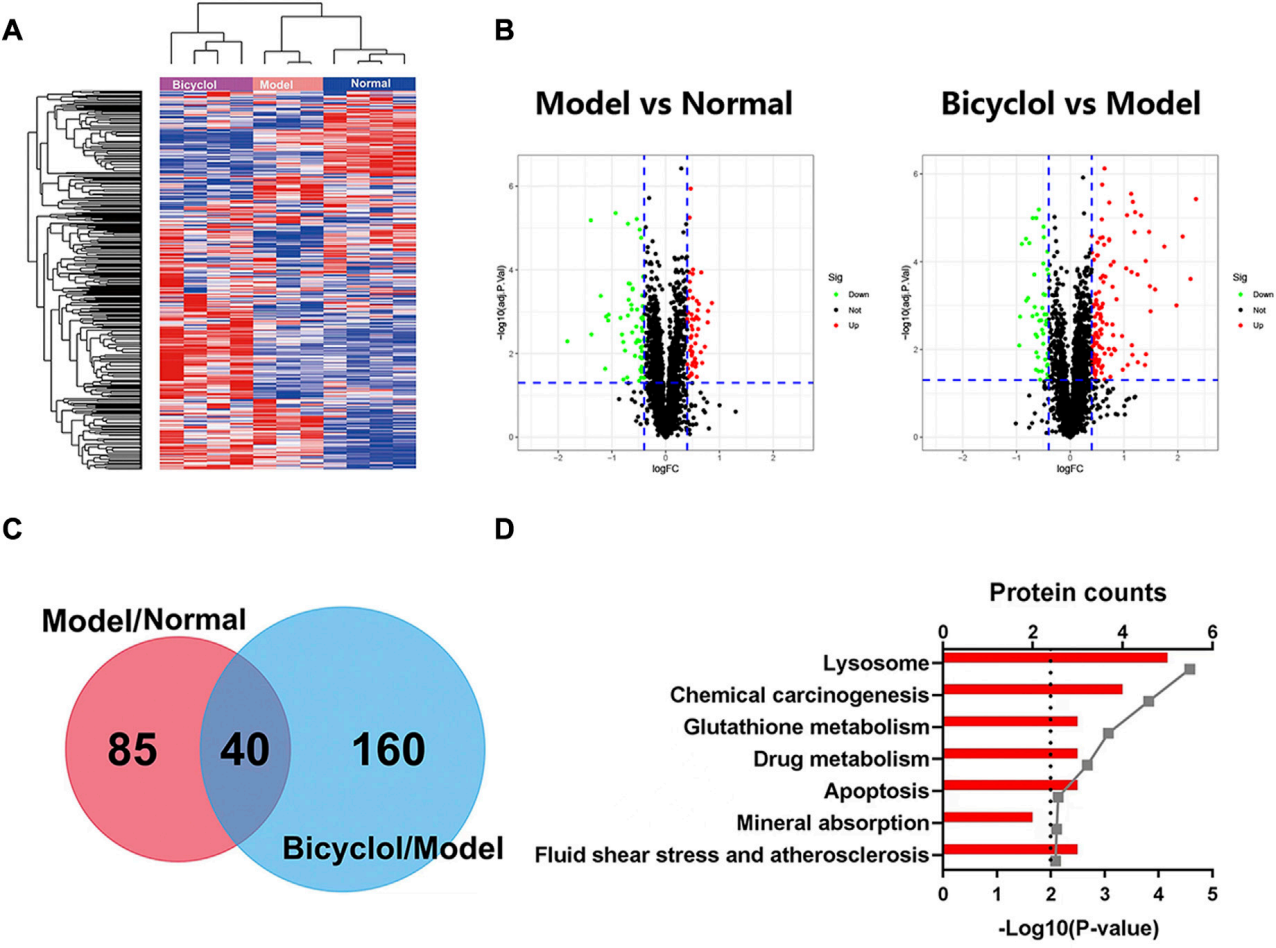
Heatmap, Volcanoplot, Venn map, KEGG pathways analyses for the changed proteins. Log 2-transformed ratio of < −0.4 or >0.4 and *p* < 0.05 were considered statistically significant. **(A)** Hierarchical clustering heat map for all the changed proteins. **(B)** Volcanoplots of the two comparisons. Green dots represent downreg ulated proteins, red dots represent upregulated proteins, black dots represent not differentially changed proteins. **(C)** Venn map of the two comparisons. **(D)** KEGG pathways analyses of 40 deregulated proteins in both Model vs Normal and Bicyclol vs Model groups.

We performed proteomics analysis for 3,926 proteins identified by one or more unique peptides to elucidate the underlying mechanisms by which bicyclol alleviated NAFLD/NASH. The scaled-down abundance of each protein in each group was as shown in Figure 3A. The expression of 125 proteins significantly changed in mice in the model group fed with HFD compared with the normal group. The expression of 200 proteins significantly changed in the bicyclol group compared with the model group. The volcano plot further revealed that bicyclol attenuated the changes in protein expression induced by HFD feeding (Figure 3B). The Venn diagram indicated that the expression of 40 proteins were significantly altered in two different comparative groups: model *versus* normal and bicyclol *versus* model (Figure 3C). The KEGG pathway analysis was used to categorize the 40 deregulated proteins. As shown in Figure 3D, the highly enriched terms included lysosome, chemical carcinogenesis, glutathione metabolism, drug metabolism, apoptosis, mineral absorption, fluid shear and atherosclerosis.

**FIGURE 4:**
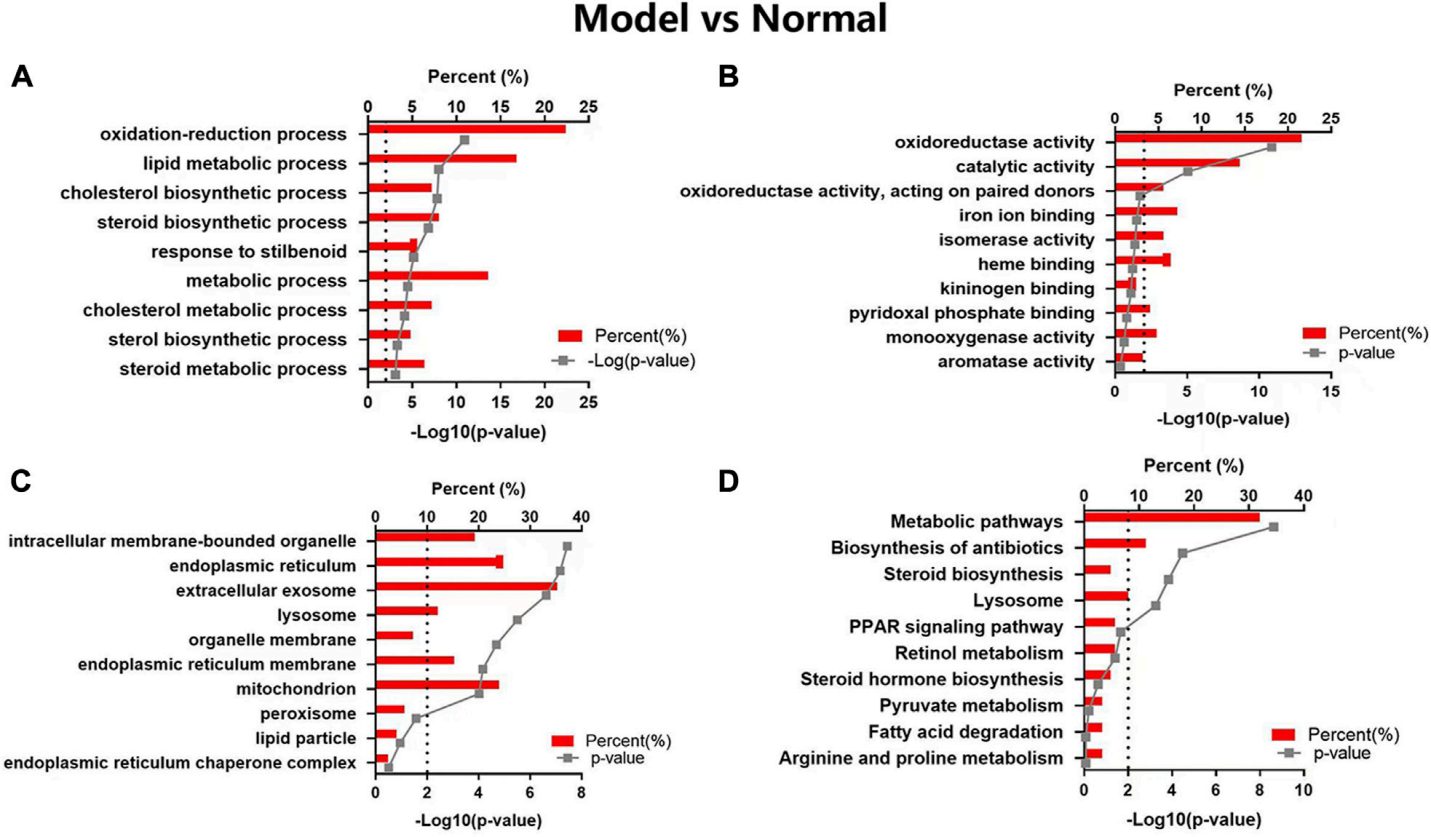
GO and KEGG pathway analyses of the changed proteins in Model vs Normal groups. The enriched items are listed according to the *p*-value. **(A)** GO-BP, **(B)** GO-MF, **(C)** GO-CC, **(D)** KEGG pathway analysis.

In the GO map, altered proteins were grouped by the DAVID functional annotation analysis. The top 10 items were enriched according to the *p* values. The GO analysis displayed that HFD feeding impacted the major pathways in biological process (Figure 4A), molecular function (Figure 4B), and cellular component (Figure 4C) related to oxidoreduction reaction and metabolic process. The KEGG pathway analysis (Figure 4D) showed that the highly enriched terms included metabolic pathways, biosynthesis of antibiotics, steroid biosynthesis, lysosome, PPAR signaling pathway, retinol metabolism, steroid hormone biosynthesis, pyruvate metabolism, fatty acid degradation, and arginine and proline metabolism.

**FIGURE 5:**
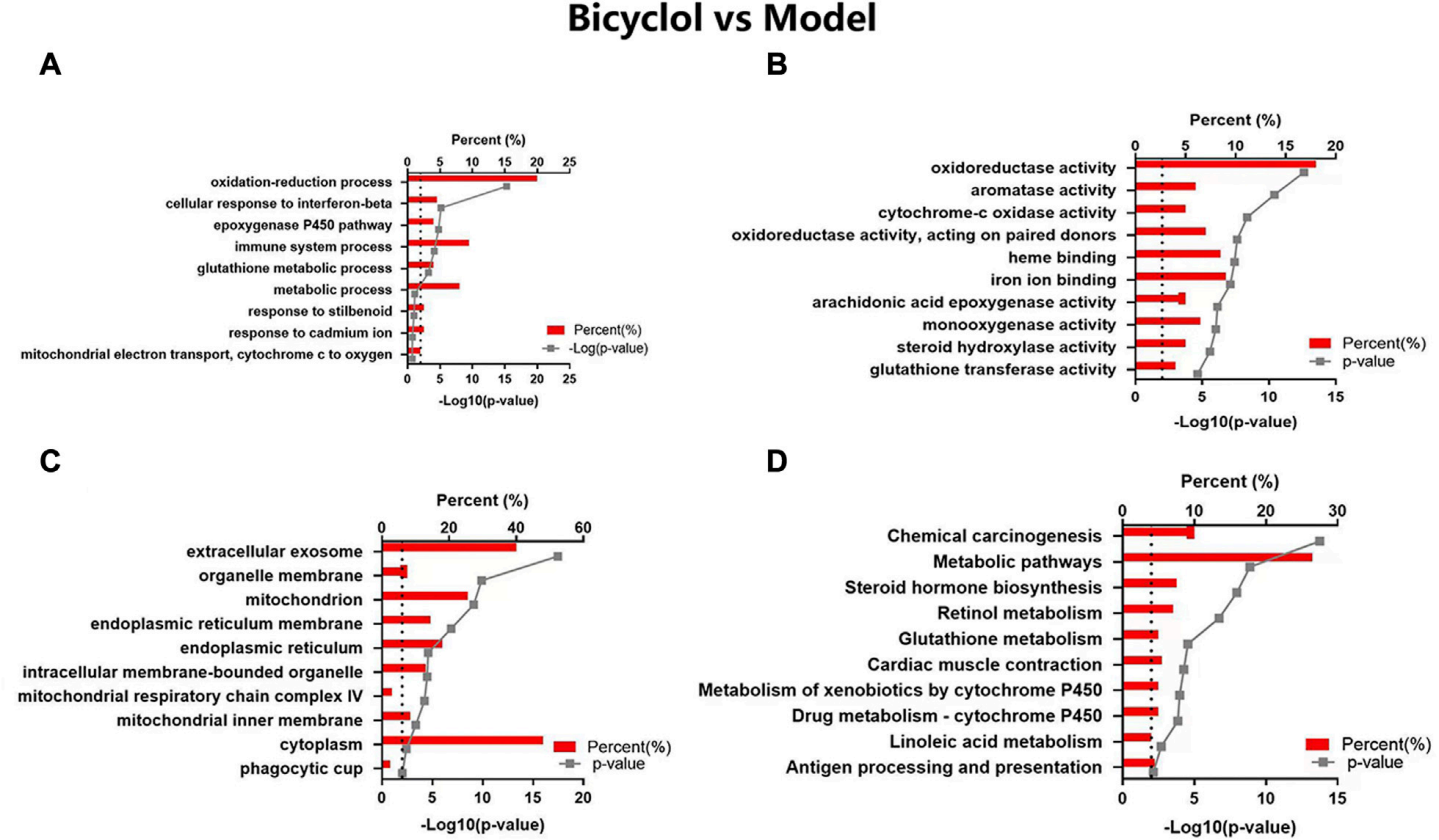
GO and KEGG pathway analyses of the changed proteins in Bicyclol vs Model groups. The enriched items are listed according to the *p*-value. **(A)** GO-BP, **(B)** GO-MF, **(C)** GO-CC, **(D)** KEGG pathway analysis.

Bicyclol could target a majority of the biological processes (Figure 5A), molecular function (Figure 5B), and cellular components (Figure 5C), such as oxidation-reduction process, epoxygenase P450 pathway, immune system process, metabolic process, iron ion binding, heme binding, mitochondrion, extracellular exosome, and monooxygenase activity. The KEGG pathway analysis (Figure 5D) revealed that the highly enriched terms included chemical carcinogenesis, metabolic pathway, steroid hormone biosynthesis, retinol metabolism, glutathione metabolism, cardiac muscle contraction, metabolism of xenobiotics by cytochrome P450, drug metabolism-cytochrome P450, linoleic acid metabolism, antigen processing and presentation.

### 3.3 Effect of bicyclol on HFD-induced inflammation and oxidative stress

**FIGURE 6:**
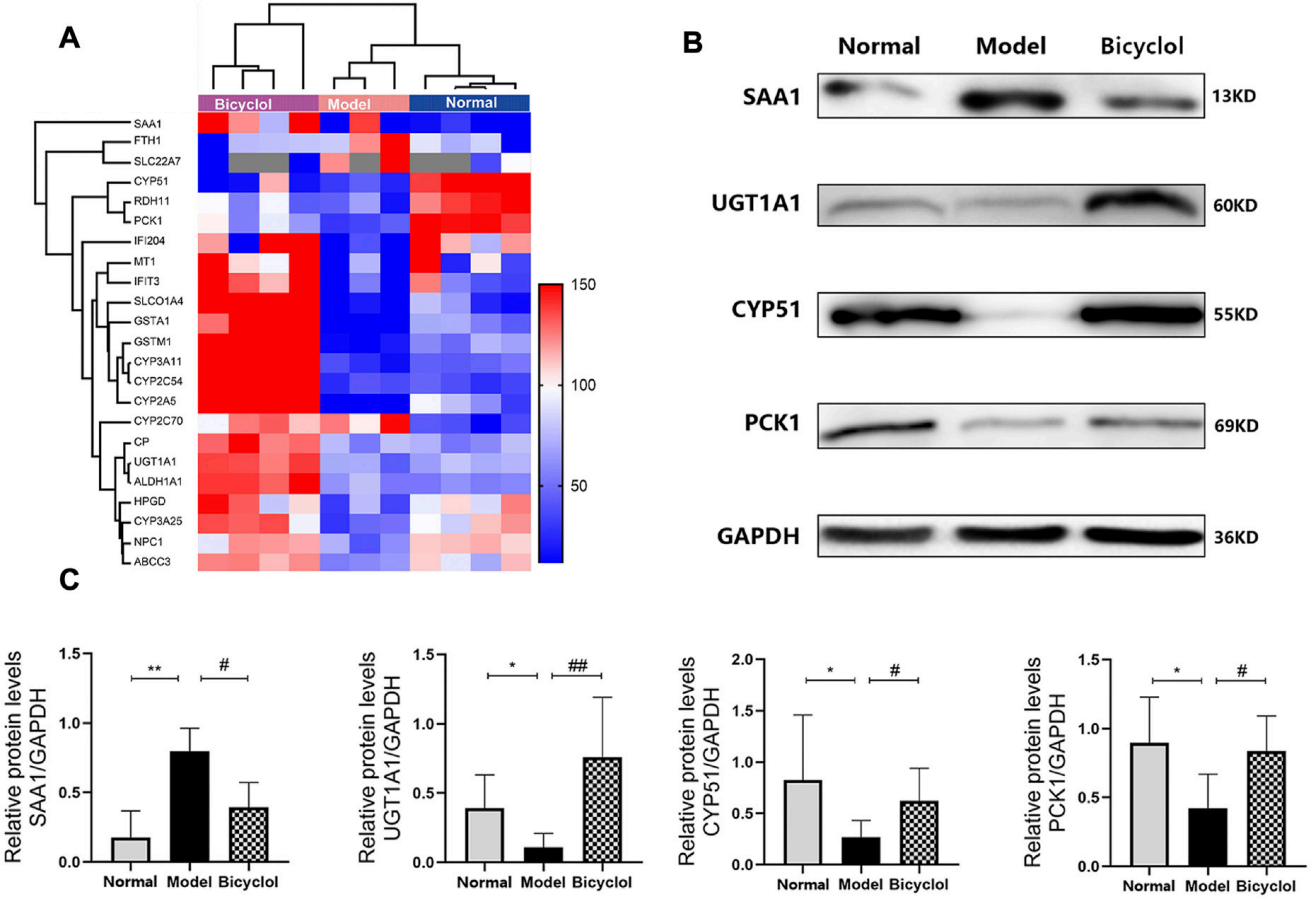
**(A)** Heat map of the proteins involved in inflammation, bile acid metabolism, cytochrome P450 pathway and biological processes. Blue and red colors indicate low and high proteins expression respectively. **(B)** Representative immunoblot images of SAA1, UGT1A1, CYP51, PCK1 and GAPDH. **(C)** Relative protein levels of SAA1 (n = 4), UGT1A1 (n = 4), CYP51(n = 4), PCK1(n = 5). *p < 0.05 vs Normal, **p < 0.01 vs Normal, #*p* < 0.05 vs Model, ##*p* < 0.01 vs Model.

Through proteomic analysis, we enriched 29 proteins that were altered in HDF-fed mice and attenuated by bicyclol treatment. The scaled-down abundance of each protein in each group was as shown in Figure 6A. The aforementioned proteins were associated with inflammation and oxidative stress, bile acid metabolism, P450 pathway, and the other biological processes.

**FIGURE 7:**
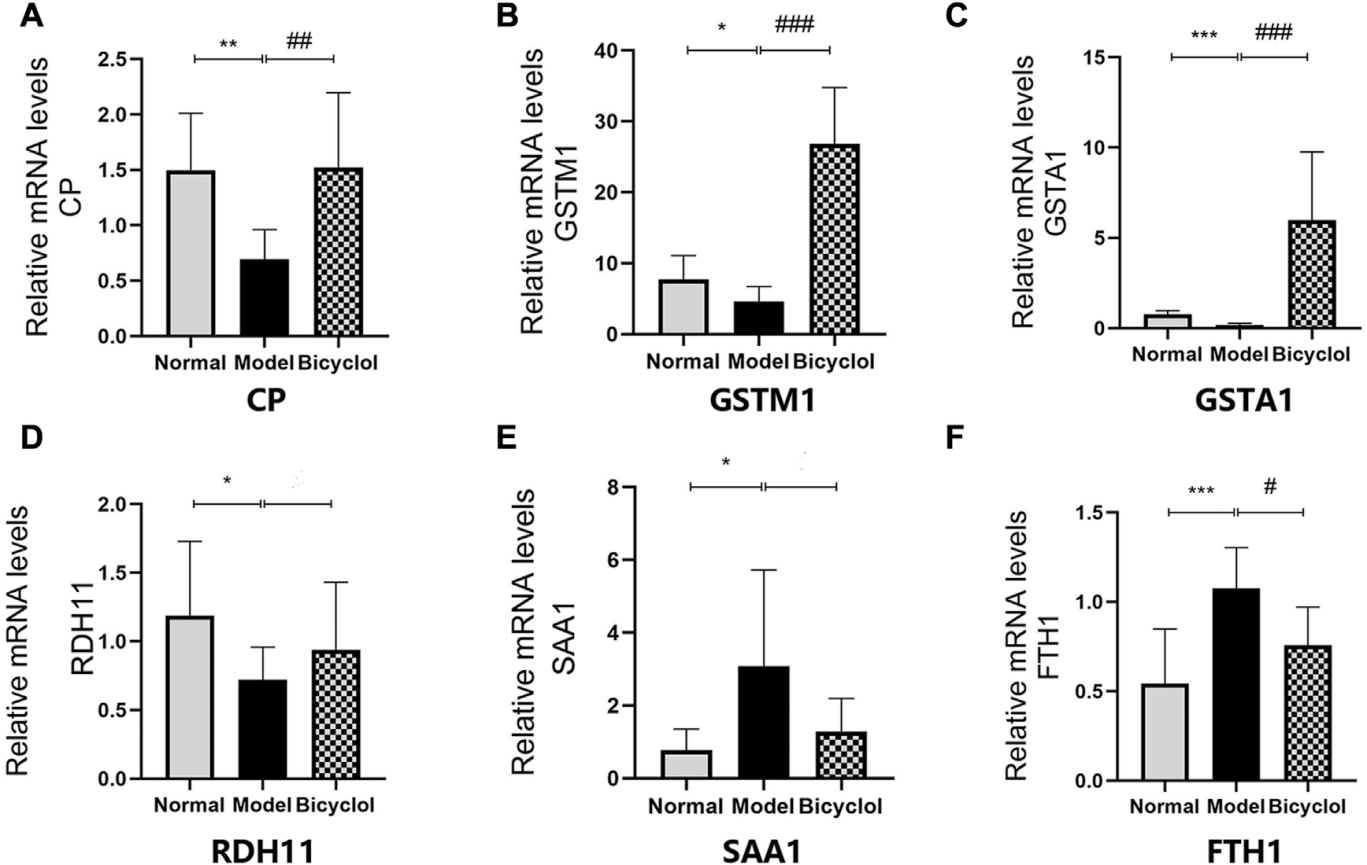
Expression of the genes involved in inflammation and oxidative stress mechanism. Relative mRNA levels of **(A)** CP (n = 8), **(B)** GSTM1 (n = 8), **(C)** GSTA1 (n = 8), **(D)** RDH11 (n = 8), **(E)** SAA1 (n = 5), **(F)** FTH1(n = 8). *p < 0.05 vs Normal, **p < 0.01 vs Normal, ***p < 0.001 vs Normal, #*p* < 0.05 vs Model, ##*p* < 0.01 vs Model, ###*p* < 0.001 vs Model.

As shown in Figure 7, RT-qPCR was performed to confirm that the HFD feeding significantly downregulated the mRNA expression levels of ceruloplasmin (CP), glutathione S-transferase Mu 1 (GSTM1), glutathione S-transferase A1 (GSTA1), and retinol dehydrogenase 11 (RDH11), and also significantly upregulated the mRNA expression levels of SAA1 and ferritin heavy chain 1 (FTH1). The altered mRNA expression levels of aforementioned genes associated with inflammation and oxidative stress were attenuated by bicyclol treatment. Using Western blot analysis, we further confirmed that the HFD feeding increased the protein levels of SAA1 which were attenuated by bicyclol treatment (Figures 6B,C).

### 3.4 Effect of bicyclol on HFD-induced bile acid metabolism

**FIGURE 8:**
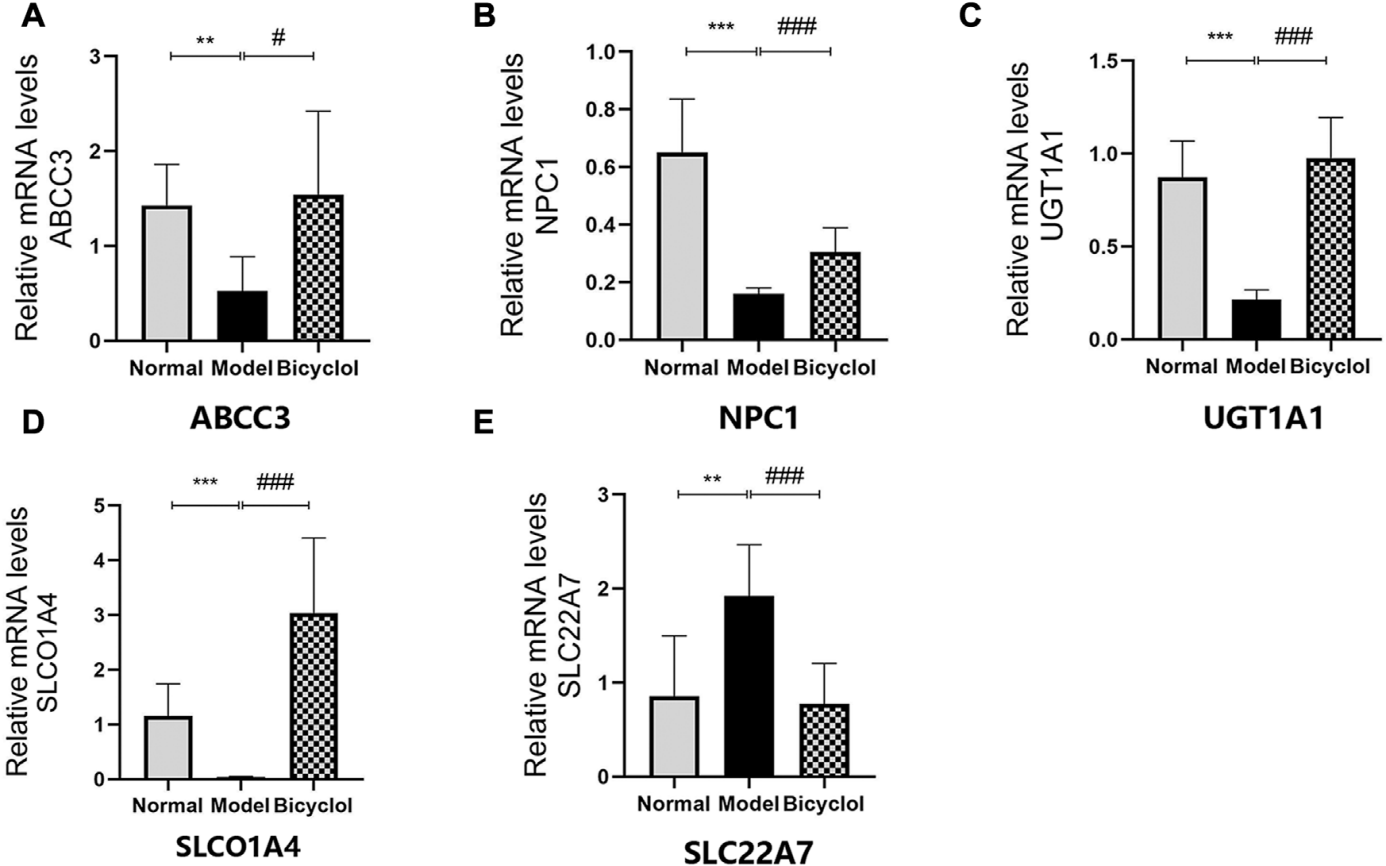
Expression of the genes involved in bile acid metabolism. Relative mRNA levels of **(A)** ABCC3 (n = 6), **(B)** NPC1 (n = 8), **(C)** UGT1A1 (n = 8), **(D)** SLCO1A4 (n = 8), **(E)** SLC22A7 (n = 8). **p < 0.01 vs Normal, ***p < 0.001 vs Normal, #*p* < 0.05 vs Model, ###*p* < 0.001 vs Model.

Proteomics analyses also showed that HFD feeding markedly changed the activation of the proteins related to bile acid metabolism such as ATP-binding cassette sub-family C member 3 (ABCC3), Niemann-Pick C1 protein (NPC1), UGT1A1, solute carrier organic anion transporter family (SLCO1A4), and solute carrier family 22 member 7 (SLC22A7), which were attenuated by bicyclel treating (Figure 6A). RT-qPCR data confirmed that the HFD feeding downregulated the mRNA levels of ABCC3, NPC1, UGT1A1, and SLCO1A4, whereas the upregulation of SLC22A7 was significantly attenuated by bicyclol treatment (Figure 8). We further confirmed the activation of UGT1A1 using Western blot analysis. As shown in Figures 6B,C, the HFD-induced decrease in UGT1A1 protein expression was attenuated by bicyclol.

### 3.5 Effect of bicyclol on HFD-induced P450 pathway

**FIGURE 9:**
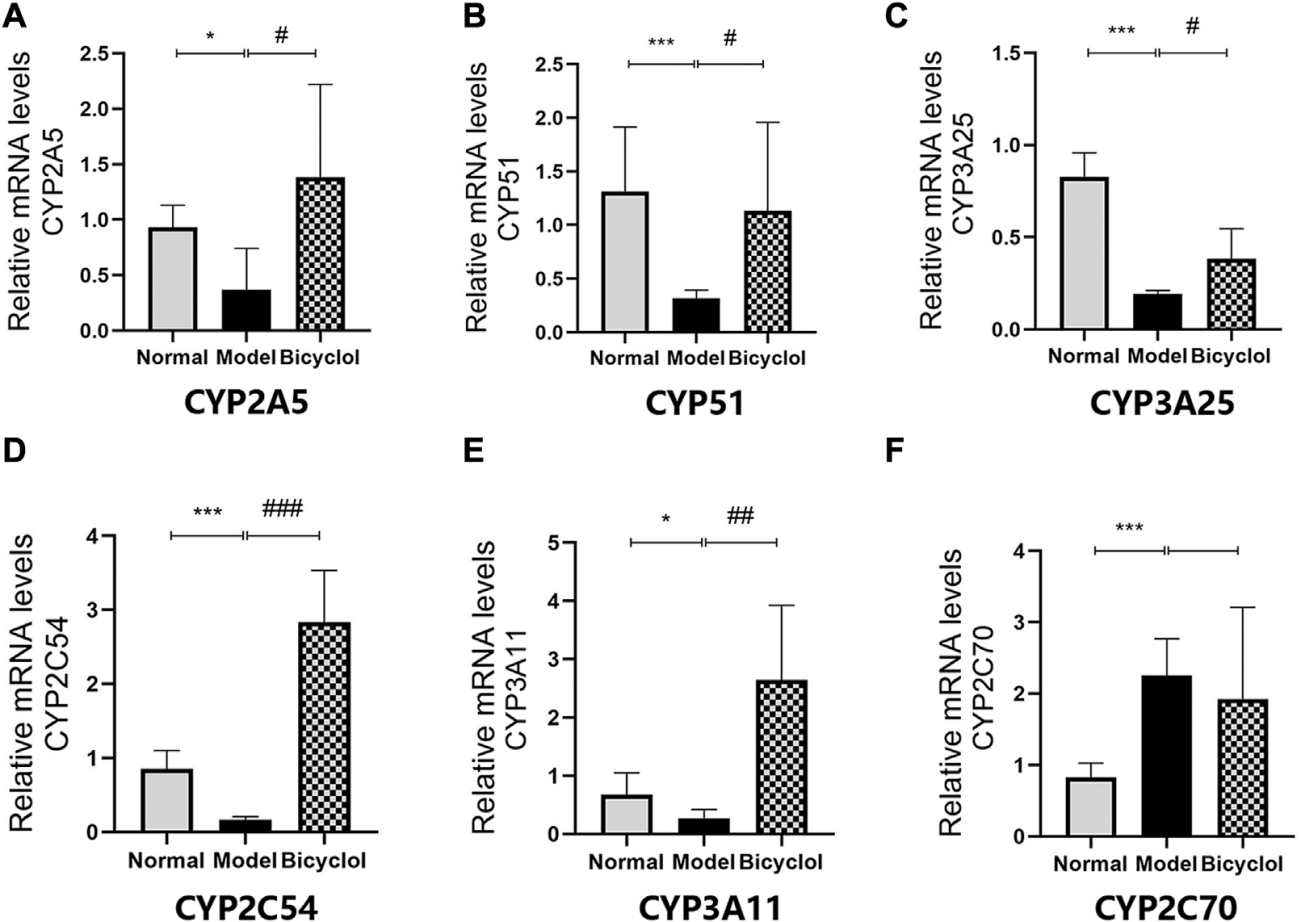
Expression of the genes involved in P450 pathway. Relative mRNA levels of **(A)** CYP2A5 (n = 5), **(B)** CYP51 (n = 8), **(C)** CYP3A25 (n = 6), **(D)** CYP2C54 (n = 8), **(E)** CYP3A11 (n = 6), **(F)** CYP2C70 (n = 5)."*p* < 0.05 vs Normal, ***p < 0.001 vs Normal, #*p* < 0.05 vs Model, ##*p* < 0.01 vs Model, ###*p* < 0.001 vs Model.

Proteomics analyses showed that HFD feeding markedly altered activation of P450 pathway, such as Cytochrome P450 2A5 (CYP2A5), CYP51, Cytochrome P450 3A25 (CYP3A25), Cytochrome P450 2C54 (CYP2C54), Cytochrome P450 3A11 (CYP3A11), and Cytochrome P450 2C70 (CYP2C70), which were attenuated by bicyclel treatment. HFD feeding downregulated the expressions of CYP2A5, CYP51, CYP3A25, CYP2C54, and CYP3A11, and upregulated the expression of CYP2C70 (Figure 6A). RT-qPCR data confirmed that the HFD feeding significantly downregulated the mRNA levels of CYP2A5, CYP51, CYP3A25, CYP2C54, and CYP3A11, whereas the upregulation of CYP2C70 was insignificantly attenuated by bicyclol treatment (Figure 9). We further confirmed the activation of CYP51 using Western blot analysis. As illustrated in Figures 6B,C, the HFD–induced decrease in CYP51 protein expression was raised by bicyclol treatment.

### 3.6 Effect of bicyclol on HFD-induced biological processes

**FIGURE 10:**
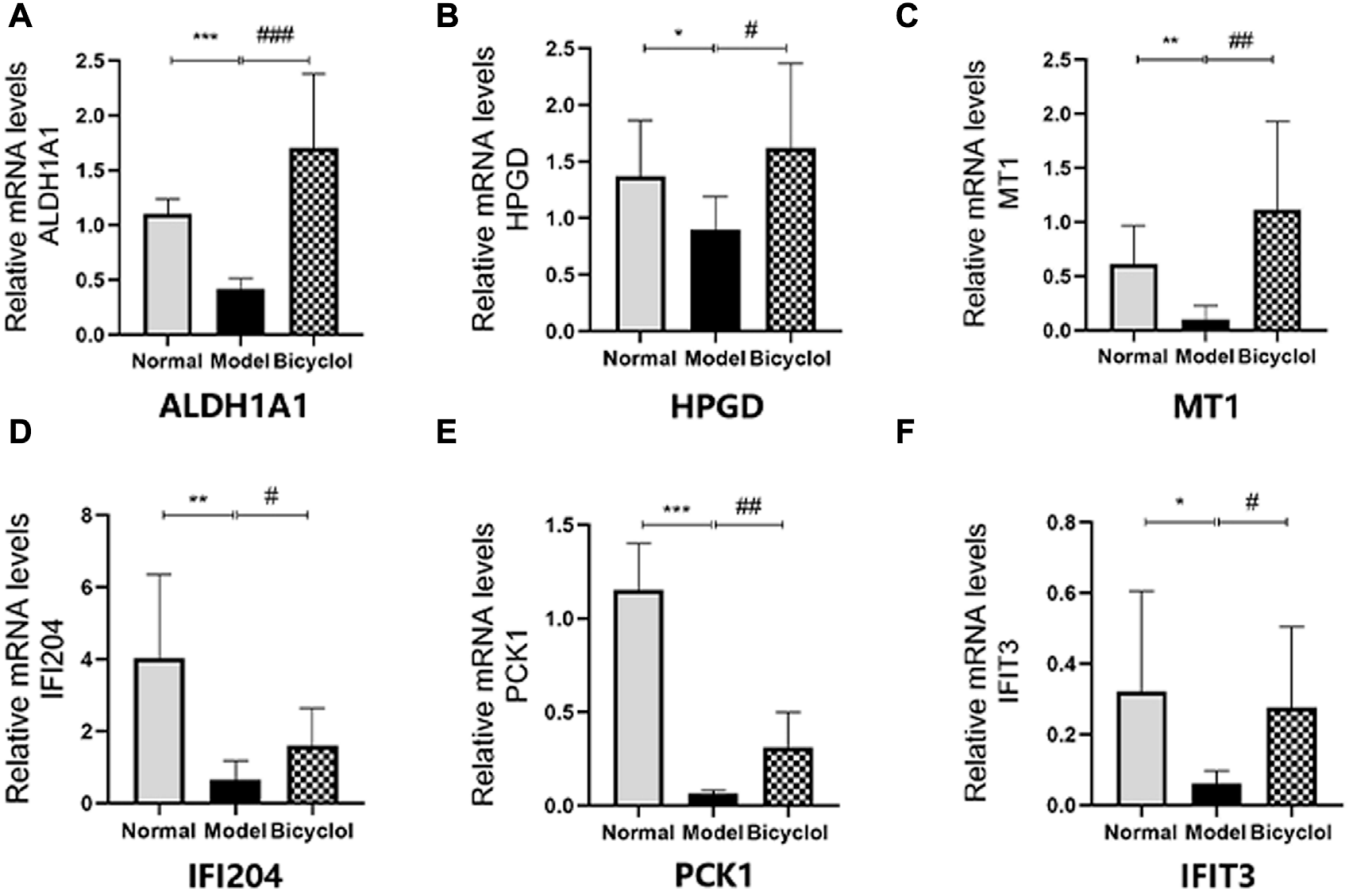
Expression of the genes involved in biological processes. Relative mRNA levels of **(A)**ALDH1A1 (n=8), **(B)** HPGD (n=6), **(C)** MT1 (n=7), **(D)** IFI204 (n=8), **(E)** PCK1 (n=6), **(F)** IFIT3 (n=8). *p < 0.05 vs Normal, **p < 0.01 vs Normal, ***p < 0.001 vs Normal, #*p* < 0.05 vs Model, ## *p* < 0.01 vs Model, ###*p* < 0.001 vs Model.

Proteomics analyses showed that the HFD feeding markedly downregulated the activation of aldehyde dehydrogenase family 1-subfamily A1 (ALDH1A1), 15-hydroxyprostaglandin dehydrogenase (HPGD), metallothionein-1 (MT1), interferon-activable protein 204 (IFI204), PCK1, interferon-induced protein with tetratricopeptide repeats 3 (IFIT3) and CP, which were attenuated by bicyclel treatment (Figure 6A). RT-qPCR data confirmed that the HFD feeding downregulated the mRNA levels of ALDH1A1, HPGD, MT1, IFI204, PCK1, IFIT3, and CP, which were significantly attenuated by bicyclol treatment (Figure 7A, Figure 10). We further confirmed the activation of PCK1 using Western blot analysis. As shown in Figures 6B,C, the HFD-induced decrease in PCK1 protein expression was raised by bicyclol treatment.

## 4 Discussion

NAFLD/NASH is a metabolic disease, which has become a major concern due to the obesity pandemic all over the world (Zeigerer, 2021). Recently, “MAFLD” (metabolic dysfunction associated with fatty liver disease) as a new terminology was suggested by some scholars to displace NAFLD (Eslam et al., 2020). More and more evidences pointed toward the “multiple-shot” hypothesis as an appropriate explanation of NAFLD pathogenesis, which is characterized by hepatic steatosis, hepatocyte damage, various degrees of fibrosis, and carcinoma (Tilg et al., 2021). In this study, inflammation injures, steato hepatitis, fibrosis, and increased activities of ALT and AST were observed with the increase in the levels of liver lipids after HFD feeding for 8 weeks.

The beneficial effects of bicyclol on inflammatory and metabolic diseases have been well documented (Li H et al., 2021). The preclinical reports and clinical studies have demonstrated that bicyclol is a potential agent for NAFLD/NASH treatment (Li et al., 2020; Zhao T et al., 2021). Most of the researches focus on one or two signaling pathway of pathogenesis in NAFLD/NASH and single target of bicyclol. However, multiple mechanisms governing the attenuating of NAFLD/NASH progress by bicyclol lack systematic investigation. In the present study, we used a Tandem Mass Tag (TMT) proteomics approach for high throughput screening protein targets of bicyclol attenuating NAFLD/NASH. Differentially expressed proteins were enriched using GO analysis and KEGG pathway, and then identified using the RT-qPCR and Western blot analyses. The results of this research provided more comprehensive insights into the potential effect of bicyclol on NAFLD/NASH progression. The proteomics analyses strongly indicated that bicyclol was an underlying therapeutic drug for NAFLD attenuating by modulating multiple pathways such as bile acid metabolism, immune response, inflammatory and oxydoredution reaction, and the P450 pathway.

Hepatic abnormal lipid accumulation can be reduced by bicyclol for targeting multiple pathways. Both animal models of NAFLD and clinical data displayed that inflammatory response was the main driving force for inducing lipid accumulation in NAFLD/NASH progression (Li et al., 2020). Many studies documented that bicyclol had a strong anti-inflammatory activity (Zhao et al., 2020; Chen et al., 2022). Based on proteomics analysis and pathway profiling, we could hypothesize that bicyclol significantly reduced HFD-induced inflammation and attenuated NAFLD/NASH progression. This study showed that HFD-feeding induced abnormal lipid accumulation and inflammation injury in hepatocyte. Bicyclol had protective effects against HFD-induced hepatic steatosis, as evidenced by the decrease in serum transaminase level, attenuation of hepatic lipids accumulation, and improvement in liver inflammatory response and fibrosis. Proteomics data, RT-qPCR and Western blot results indicated that bicyclol was able to attenuate HFD-induced activation of inflammation and oxidative stress.

Consistent with the results of this study, the expression levels of SAA1, as an acute-phase apolipoprotein reactant under the regulation of inflammatory cytokines, were elevated in liver tissues from mice with HFD-induced steatosis (Li D et al., 2021). SAA1 triggered hepatic steatosis and regulated inflammatory response by forming a SAA1/TLR4/NF-κB feedforward, which leads to NAFLD progression (Jiang et al., 2022). Also, CP was a positive acute-phase reactant and its levels increased in inflammatory states or cell damage (Wang and Wang, 2019). Both RT-qPCR and Western blot data showed that bicyclol treatment significantly improved the upregulation of SAA1 and CP induced by HFD feeding. The GO analyses showed that the oxidation-reduction, immune system process, metabolic process and P450 pathway were the top biological processes altered during NAFLD/NASH progression, which were attenuated by bicyclol treatment.

As a principal cop binding protein, Cp also played a vital role in holding hepatic copper homeostasis by regulating the transport of copper. A present study revealed that CP might be a target to ameliorate NAFLD development by modulating copper-SCO1-AMPK signaling pathway (Xie et al., 2022). Therefore, MT1 plays a crucial role in inflammatory and immunity diseases by acting as an intracellular iron transport agent (Dai et al., 2021). IFI204 and IFIT3 have biological roles in innate immune responses for bacterial and viral infection (Chunfa et al., 2017; Ciccone et al., 2018). ALDH1A1 is associated with the tumour microenvironment by promoting angiogenesis (Ciccone et al., 2018). The proteomic and RT-qPCR results indicated that the metal ion metabolism, immune response and angiogenesis may represent several underlying molecular mechanisms of bicyclol ameliorating NAFLD/NASH. Therefore, it is reasonable to presume that metal ion metabolism such as ferroptosis and cuproptosis may be involved in the pathogenesis of NAFLD/NASH and acting mechanism of bicyclol.

In addition, the effects of bicyclol in regulating cytochrome P450 enzymes activities in hepatic microsomes were important mechanism for NAFLD/NASH progression. Pharmacokinetic study verified that the substrate of CYP3A/2E1 enzyme was bicyclol (Zhao T et al., 2021). Genetic analyses displayed that the activity of the enzyme CYP3A11 was significantly altered in NAFLD mice (Lad et al., 2022). The Cyp3a25/Cyp2b10 pathway could regulate hepatocyte apoptosis (Wu et al., 2022). The data indicated that bicyclol markedly upregulated the mRNA expression of both CYP3A11 and CYP3A25. We predicted that the activation of CYP3A11 and CYP3A25 by bicyclol might be beneficial in NAFLD/NASH treatment. Moreover, CYP51 is a super family of cytochrome P450 enzymes that plays an important role in sterol and cholesterol biosynthesis (Yin et al., 2022). As illustrated in Figure 6C, Figure 9B, the HFD feeding downregulated both mRNA and protein expression levels of CYP51, which were attenuated by bicyclol. Hence, the P450 enzymes such as CYP3A11, CYP3A25, and CYP51 might serve as new targets for improving NAFLD/NASH.

Another potential protective mechanism of bicyclol is the altered bile acid metabolism. UGT1A1 is a highly polymorphic enzyme responsible for detoxification and metabolic clearance of the endogenous toxin bilirubin (Zhu et al., 2020). The reduced activity/expression of UGT1A1 may elevate the levels of serum bilirubin and lead to drug-induced liver damage (Hulshof et al., 2022). As shown in Figure 6C, Figure 8C, the HFD feeding downregulated both mRNA and protein expression levels of UGT1A1, which were ameliorated by bicyclol. Therefore, the findings of this study predicted that inhibitor of UGT1A1 might be helpful for NAFLD/NASH treatment. As the bile acid pathway mediated genes, ABCC3 played a central role in bile acid transporter and NPC1 maintained cholesterol homeostasis (Banach et al., 2022; Lu, 2022). The mRNA levels of both ABCC3 and NPC1 were reduced in the liver tissues of mice fed with HFD. The aforementioned proteins all have potential as new targets for improving NAFLD/NASH. However, the detailed molecular mechanisms involved in this pathway are still unclear.

In conclusion, this study provided comprehensive information for elucidating the molecular mechanisms of bicyclol as an underlying preventive agent for NAFLD/NASH. Bicyclol could target multiple pathways related to inflammation, oxidative stress, bile acid metabolism, metal ion metabolism, immune response and angiogenesis. This study indicated that bicyclol might be worth of further clinical research.

## Data Availability

The datasets presented in this study can be found in online repositories. The names of the repository/repositories and accession number(s) can be found below: ProteomeXchange via the PRIDE database; PXD040233.
