# Erratum: Bicyclol attenuates high fat diet-induced non-alcoholic fatty liver disease/non-alcoholic steatohepatitis through modulating multiple pathways in mice

**DOI:** 10.3389/fphar.2023.1204546

**Published:** 2023-04-20

**Authors:** Frontiers Production Office

**Affiliations:** Frontiers Media SA, Lausanne, Switzerland

**Keywords:** bicyclol, NAFLD, NASH, proteomics, multiple pathways

Due to a production error, an author name was incorrectly spelled as “Benghong Xu”. The correct spelling is “Benhong Xu”. The publisher apologizes for this mistake.

The original version of this article has been updated.

